# Unraveling heterogeneity and treatment of asthma through integrating multi-omics data

**DOI:** 10.3389/falgy.2024.1496392

**Published:** 2024-11-05

**Authors:** Wei Zhang, Yu Zhang, Lifei Li, Rongchang Chen, Fei Shi

**Affiliations:** ^1^ Department of Infectious Diseases, the First Affiliated Hospital (Shenzhen People’s Hospital), School of Medicine, Southern University of Science and Technology, Shenzhen, China; ^2^ Department of Infectious Diseases, Shenzhen People’s Hospital (The First Affiliated Hospital, Southern University of Science and Technology; The Second Clinical Medical College, Jinan University), Shenzhen, China; ^3^ Key Laboratory of Shenzhen Respiratory Diseases, Institute of Shenzhen Respiratory Diseases, Department of Respiratory and Critical Care Medicine, Shenzhen People’s Hospital (The First Affiliated Hospital, Southern University of Science and Technology; The Second Clinical Medical College, Jinan University), Shenzhen, China

**Keywords:** asthma, heterogeneity, multi-omics, patient stratification, treatment

## Abstract

Asthma has become one of the most serious chronic respiratory diseases threatening people's lives worldwide. The pathogenesis of asthma is complex and driven by numerous cells and their interactions, which contribute to its genetic and phenotypic heterogeneity. The clinical characteristic is insufficient for the precision of patient classification and therapies; thus, a combination of the functional or pathophysiological mechanism and clinical phenotype proposes a new concept called “asthma endophenotype” representing various patient subtypes defined by distinct pathophysiological mechanisms. High-throughput omics approaches including genomics, epigenomics, transcriptomics, proteomics, metabolomics and microbiome enable us to investigate the pathogenetic heterogeneity of diverse endophenotypes and the underlying mechanisms from different angles. In this review, we provide a comprehensive overview of the roles of diverse cell types in the pathophysiology and heterogeneity of asthma and present a current perspective on their contribution into the bidirectional interaction between airway inflammation and airway remodeling. We next discussed how integrated analysis of multi-omics data via machine learning can systematically characterize the molecular and biological profiles of genetic heterogeneity of asthma phenotype. The current application of multi-omics approaches on patient stratification and therapies will be described. Integrating multi-omics and clinical data will provide more insights into the key pathogenic mechanism in asthma heterogeneity and reshape the strategies for asthma management and treatment.

## Introduction

Bronchial asthma is one of the most common chronic inflammatory diseases, characterized by three hallmark features: airway remodeling, airway inflammation, and airway hyperresponsiveness. With a global prevalence exceeding 300 million cases, including a notable 60 million in China, the morbidity and mortality rates of asthma are increasing sharply. This escalation, along with the disease's complications, imposes a substantial economic burden worldwide (1). Individuals with asthma exhibit significant variability in pathology, severity, phenotype, and therapeutic responses. Consequently, asthma can be classified into different subtypes based on distinct physiological and clinical features. Allergic asthma, one major phenotype, accounts for 60%–80% of cases and is often associated with a family history of allergic diseases (2). This form of asthma is primarily driven by a T helper 2 cells (Th2) immune response, with inhaled corticosteroids (ICS) being the first-line treatment (3). However, approximately 10% of patients with severe asthma remain poorly controlled despite high doses of ICS, oral corticosteroids, and other treatments such as theophylline (4). Asthma's heterogeneity is predominantly driven by interactions between genetic and environmental factors, with multiple cell types and their interactions contributing to its complexity. The interplay between airway inflammation and airway remodeling remains largely unclear but is considered central to asthma's progression and severity. Over the past decades, extensive omics data including genomics, epigenomics, transcriptomics, proteomics, metabolomics, and microbiome studies have been generated for asthma research. These datasets provide a comprehensive characterization of the molecular and biological profiles underlying the genetic heterogeneity of asthma phenotypes and facilitate the identification of potential biomarkers and therapeutic targets from multiple perspectives. In this review, we examine the genetic and phenotypic heterogeneity of asthma, with a focus on the roles of various cell types and their interactions in contributing to asthma pathology. We emphasize the application of multi-omics approaches to unravel the complex and diverse pathogenesis of asthma heterogeneity and to explore novel therapeutic strategies.

## Updating the roles of key cell types in asthma pathophysiology and heterogeneity

**Figure 1:**
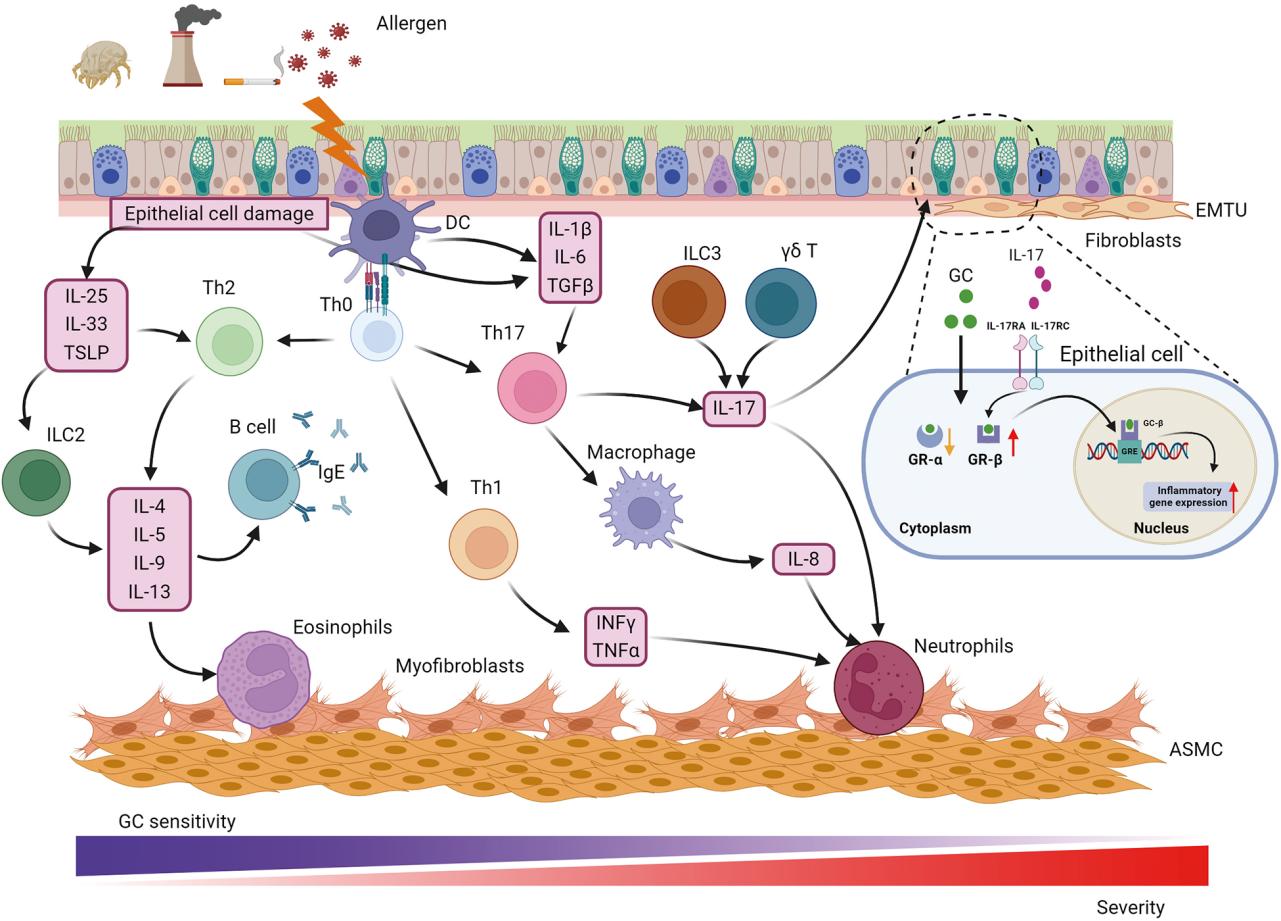
Pathophysiology of asthma. Asthma is a heterogeneous disease with various cells, cytokines and chemokines involved in its pathogenesis. Allergens such as respiratory pathogens and pollutants induce epithelial barrier dysfunction and initiate immune response through IL-25, IL-33 and TSLP to promote Th2-cell differentiation while IL-1β, IL-6, TGFβ from epithelial cells and DCs participate in Th17-cell development. Th2 and Th17 mediate eosinophilic and neutrophilic inflammation via the synthesis of a Th2 cytokine profile (IL-4, IL-5, IL-9, and IL-13) and IL-17, respectively. In addition, IFNγ and TNFα from Th1-cell contribute to neutrophilic inflammation. Except for neutrophilic infiltration, Th17 cells also play a central role in activating macrophage polarization and IL-8 release. In addition to Th17, IL-17 can be derived from ILC3 and γδ T cell, which is involved in GC resistance, airway remodeling and hyperresponsiveness through binding to IL-17RA/IL-17RC on epithelial cells and up-regulating GR-β. The main cause that neutrophilic patients poorly response to GC therapy could be higher expression of GR-β and lower expression of GR-α. The communication between epithelial and fibroblast forms the EMTU contributing to airway remodeling, airway inflammation and GC insensitivity. Myofibroblast was considered a cell resource for increased ASMC. ASMC, airway smooth muscle cell; EMTU, epithelial-mesenchymal trophic unit; GC, glucocorticosteroid; GR, GC receptor; GRE, GC response elements; IFNγ, interferon γ; IL-, interleukin; IL-17 RA/RC, IL-17 receptor A/C; ILC2, 2 innate lymphoid cells; ILC3, 3 innate lymphoid cells; TGFβ, transforming growth factor β; Th, T helper cells; TNFα, tumor necrosis factor α; TSLP, thymic stromal lymphopoietin.

Progression of airway inflammation and airway remodeling in asthma is a multistep process influenced by numerous cells and their interactions (Figure 1). Specifically, key inflammatory cells and immunological factors, such as T cells, eosinophils, dendritic cells (DCs), and proinflammatory cytokines, play significant roles in the allergic manifestations of asthma. Particularly, a disruption in the Th1/Th2 balance has been implicated in the onset of asthma. Patients with asthma are typically categorized into “Th2” or “non-Th2” phenotypes (5). Distinct from Th2 asthma, non-Th2 asthma can be further stratified into neutrophilic, mixed, and paucigranulocytic phenotypes based on the cellular composition in sputum specimens (5). Th2 inflammation, the most common form of asthma, involves a systematic allergic response comprising a sensitization phase and an effector phase (6). During the sensitization phase, allergens and pathogens crossing the epithelial barrier are phagocytosed by DCs, which respond to cytokines produced by epithelial cells. DCs present allergen-derived peptides via major histocompatibility complex (MHC)-II molecules to CD4+ T cells, collaborating with basophils to drive the differentiation of these T cells into Th2 cells. Increasing evidence suggests that epithelial-derived cytokines, including IL-33, thymic stromal lymphopoietin (TSLP), and IL-25, act as master regulators mediating both innate and adaptive immunity. Type 2 innate lymphoid cells (ILC2) are activated by these epithelial cell-derived cytokines, inducing the production of interleukin (IL)-5, IL-9, and IL-13, which further activate eosinophils and promote Th2 airway inflammation (7). Th2 cells and T follicular helper cells secrete IL-4, IL-13, and IL-21, promoting B-cell maturation and differentiation into plasma cells that produce immunoglobulin (Ig) E. This IgE binds to the IgE receptor (FcεRI) on mast cells and basophils, leading to organismal sensitization. Th2 and Th17 cells mediate eosinophilic and neutrophilic inflammation via the synthesis of Th2 cytokines (IL-4, IL-5, IL-9, and IL-13) and IL-17, respectively. In addition, IL-4 and IL-13 could induce macrophage polarization, which further contributes to the complexity and heterogeneity of asthma pathogenesis (8). During the effector phase, allergen cross-linking of FcεRI-bound IgE molecules through the epithelial barrier triggers degranulation of mast cells and basophils. This results in the rapid release of various inflammatory mediators, including leukotrienes, histamine, prostaglandins, and platelet-activating factors, culminating in smooth muscle cell contraction and mucus secretion.

Specifically, immune cells and their releasing cytokines are the key influencers tightly associated with airway inflammation and asthma severity as well as heterogeneity. For instance, IFNγ and TNFα from Th1 cells contribute to neutrophilic inflammation in not Th2 asthma (9). Besides, numerous studies have shown that the expression level of IL-17 is frequently elevated in non-Th2 asthma and is strongly associated with severe asthma. IL-17 facilitates the recruitment of neutrophils and promotes the production of transforming growth factor β (TGFβ), which amplifies the inflammatory response and contributes to airway remodeling in severe asthma (10). Additionally, IL-17 has been demonstrated to enhance the expression of glucocorticosteroid receptor β (GR-β) in peripheral blood mononuclear cells and epithelial cells from asthmatic patients with steroid resistance (11, 12). IL-17 correlated with glucocorticoid resistance through binding to IL-17RA/IL-17RC on epithelial cells and up-regulating GR-β, which refer to the primary reason for poor response to glucocorticoid therapy in neutrophilic patients. While Th17 cells are considered the primary source of IL-17, recent studies have shown that IL-17 can also be derived from type 3 innate lymphoid cells (ILC3) and γδ T cells (13–15). Innate lymphoid cells (ILCs) are a newly discovered class of innate immune cells that are widely present in the lungs. Unlike T cells, which require specific T cell receptor activation, ILCs lack specific surface receptors and respond directly to local stimuli at the lung epithelial barrier, rapidly exerting their effects through cytokine production (16). ILCs are divided into three major subgroups: ILC1, ILC2, and ILC3. Among these, ILC2 can produce Th2 cytokines such as IL-4, IL-5, and IL-13, which made them not only initiate airway inflammation but also orchestrate the recruitment and activation of other innate and adaptive immune cells, further amplifying the inflammatory response (17). Moreover, ILC2s exhibit substantial cytokine plasticity, as they also produced Th1 or Th17 under appropriate conditions, highlighting their profound contribution to not Th2 asthma, such as neutrophilic asthma. Moreover, ILCregs are another subset of innate lymphoid cells that could contribute to the resolution of allergic airway inflammation through IL-10 production (18). IL-10 is an anti-inflammatory cytokine that plays a vital role in modulating the immune response to prevent excessive tissue damage and promote the restoration of homeostasis (19). Recent studies have shown that ILC2s exhibit plasticity and can also produce IL-10 (20). This plasticity allows ILC2s to adapt their functions based on the local microenvironment, which can be crucial during different phases of asthma, such as in the presence of chronic inflammation or during resolution (21). Thus, to investigate the mechanisms of ILC2 functions and their regulators involved in diverse asthma phenotypes will facilitate patient stratification and precise personal therapies.

In asthma, structural cells play a critical role in the airway remodeling as well as in airway inflammation. The structural changes in the airways of asthmatic patients primarily include epithelial damage, goblet cell hyperplasia, subepithelial fibrosis, reticular basement membrane thickening, and airway smooth muscle hypertrophy (22). The airway epithelium serves as a frontline defense and central player in the pathogenesis of asthma, initiating early immune responses and defense mechanisms against pathogens. Epithelial cells release a wide range of chemokines, inflammatory cytokines, and other regulators to activate and recruit immune cells in response to external stimuli. For instance, interferons (IFNs) such as IFNλ and IFNβ produced by the epithelium play a crucial role in antiviral responses and viral clearance (23). Moreover, the epithelium was key component contributing to airway remodeling. Mucins 5AC (MUC5AC) and MUC5B, produced by the epithelium, are major components of airway mucus. Overproduction of MUC5AC and decreased secretion of MUC5B have been observed in the sputum of asthmatic individuals. Increased MUC5AC is especially associated with goblet cell hyperplasia and an elevated risk of asthma (24). Chloride channel regulator 1 (CLCA1) is involved in promoting mucus secretion and regulating innate immune responses in asthma. However, the precise mechanisms by which CLCA1 modulates mucus production and innate immunity remain unclear (25).

Recently, single-cell technologies have been able to identify cell interactions playing a role in the interplay of airway inflammation and airway remodeling in asthma. For instance, the frequent communication between epithelial cells and fibroblasts forms an epithelium-mesenchymal trophic unit (EMTU), which mediates the asthmatic microenvironment and drives pathogenic remodeling (26). Epithelial and inflammatory cell-derived bone morphogenetic proteins and TGFβ can activate fibroblasts, causing their differentiation into myofibroblasts. These myofibroblasts perform multiple functions involved in airway inflammation and remodeling, including generating contractile force, producing and depositing extracellular matrix components, and secreting cytokines (27). Although the relationship between myofibroblasts and airway smooth muscle cells (ASMCs) remains unclear, numerous studies suggest that fibroblast-myofibroblast transition may provide precursor cells for generating ASMCs, as myofibroblasts have been found to migrate from the epithelium towards the smooth muscle mass in patients with mild asthma (28, 29). The studies of the relationship between airway inflammation and airway remodeling are ongoing, with evidence suggesting that airway remodeling can be either dependent on or independent of airway inflammation. Traditionally, studies using tissue biopsies and animal models have indicated that airway remodeling is a possible secondary event to airway inflammation, strongly correlated with disease severity (30, 31). However, airway cross-section measurements in patients with asthma have shown increased airway smooth muscle mass even in the absence of inflammation (32, 33). With the advancement of sequencing technologies, a recent study has identified epithelial-derived IL-33 as a hallmark of severe asthma (34). IL-33 can exacerbate both airway inflammation and airway remodeling by promoting macrophage polarization and fibroblast activation, suggesting a bidirectional interaction between these processes (35). Furthermore, a previous study has found interaction between macrophage and fibroblast promoting the airway inflammation and airway remodeling in asthma, suggesting that crosstalk between immune cells and structural cells might play a crucial role in the parallel progression of airway inflammation and remodeling (13). These findings indicate that targeting the disruption of cell interplay could be a novel therapeutic strategy for asthma. Investigating cell-cell communication and its contribution to the phenotypic and genetic heterogeneity of asthma is essential for elucidating the relationship between airway inflammation and remodeling. This knowledge will advance precision medicine approaches for asthma, potentially leading to improved therapeutic outcomes.

## Multi-omics approaches have emerged as essential tools for investigating asthma pathology and heterogeneity

Traditionally, asthma patients have been categorized into various subtypes based on clinical features such as exacerbating factors (allergens, exercise, and infections), age of onset, comorbidities (sinusitis and obesity), and response to treatment (36–38). According to disease severity, asthma can be classified into four categories: (1) intermittent asthma, (2) mild persistent asthma, (3) moderate persistent asthma, and (4) severe persistent asthma (39). Regarding the age of onset, asthma is classified into two main categories: early-onset asthma and late-onset asthma (40). However, while these classifications based on clinical phenotypes provide valuable insights into persistent symptoms, physiological abnormalities, and inflammatory mechanisms of asthma, they have limitations in fully capturing the heterogeneity of the disease (41).

Currently researchers have introduced a novel concept known as “asthma endophenotype”, which combines functional or pathophysiological mechanisms with clinical phenotype to better understand the disease (38, 42). For instance, corticosteroid-dependent asthma has been categorized into two distinct inflammatory groups based on the presence or absence of eosinophils in endobronchial biopsies and lavages (38). Based on the predominant immune-inflammatory pathways, asthma endotypes are commonly classified into three main categories: high Th2 type, low Th2 type, and mixed endotype (43). The Th2-high endotype is characterized by eosinophilic airway inflammation, while the Th2-low endotype typically exhibits neutrophilic or oligogranulocytic airway inflammation. Sputum cytometry is currently considered the most clinically valid method for quantitatively and reactively assessing airway inflammation. Sputum analysis by flow cytometry categorizes asthma into four groups: eosinophils, neutrophils, mixed granulocytes (a transition phenotype with increased proportions of both eosinophils and neutrophils), and paucigranulocyte (neither eosinophils nor neutrophils elevated) (44). Compared to eosinophilic and neutrophilic asthma, patients with mixed granulocytic asthma experience higher rates of exacerbation, more severe airflow obstruction, and daily wheezing. In order to elucidate the pathogenesis of diverse endophenotype of asthma, establishing appropriate animal models that represent the typical pathophysiology of asthma is crucial. The most commonly used allergens for establishing murine models of asthma are ovalbumin (OVA) and house dust mite (HDM), which can induce Th2-high or Th2-low asthma, respectively (45). Additionally, high and low doses of lipopolysaccharide (LPS) combined with OVA or HDM can be used to establish murine models representing neutrophilic and mixed-granulocytic asthma, respectively (46). These models allow researchers to investigate pathological mechanisms and evaluate the efficacy of therapeutic interventions, as well as mechanisms underlying drug resistance and treatment response.

The complex pathogenesis of asthma involves diverse cascades of events across various omics levels, and the utilization of high-throughput omics approaches has become increasingly prominent in asthma research over the past decades. The advent of the Human Genome Project has led to the availability of vast amounts of omics data in open resources (47), encompassing genomics for risk variant detection, epigenomics for the study of gene dysregulation, transcriptomics for the identification of differential gene expression, proteomics for the detection of abnormal translation, immunome analysis for therapeutic target determination, metabolomics for metabolite profiling, and microbiome and exposome analysis for characterizing subtype-specific environmental exposures. These omics data enable the screening of numerous genes, transcripts, proteins, and metabolites for potential biomarkers in an unbiased manner (48). The integration of multi-omics approaches has significantly enhanced our understanding of asthma phenotypes and the underlying cellular processes and molecular functions. Moreover, these technologies provide valuable tools for precise patient stratification, which is crucial for improving asthma management strategies. Overall, the application of high-throughput omics techniques has revolutionized asthma research, offering insights into its complex etiology and paving the way for more targeted and personalized approaches to asthma diagnosis, treatment, and management.

Asthma results from a complex interplay between environmental and genetic factors, with epigenetic regulation serving as a critical mediator of the interaction between genes and the environment, independent of changes in DNA sequence. Among the various mechanisms of epigenetic regulation, DNA methylation and histone modification are particularly implicated in the pathogenesis and progression of asthma. DNA methylation, mediated by DNA methyltransferases DNMT1, DNMT2, and DNMT3 (49), commonly occurs in CpG islands associated with promoters and other cis-regulatory elements, thereby impeding transcription factor binding and subsequently silencing gene expression. Studies have identified PM2.5 exposure as a factor that enhances DNA methylation levels, particularly at the promoter of ten-eleven translocation methylcytosine dioxygenase 1 (TET1), which has been linked to childhood asthma onset and traffic-related air pollution (50). Epigenome-wide association studies have explored the relationship between asthma and alterations in DNA methylation across the entire genome, pinpointing differentially methylated loci in genes such as IL-5 receptor alpha and potassium voltage-gated channel subfamily H member 2 (KCNH2) as potential biomarkers for asthma risk and targets for treatment strategies (51). Similarly, histone modification, akin to DNA methylation, influences transcriptional activity without altering the DNA sequence. Acetylation, methylation, ubiquitination, and phosphorylation are common types of histone modifications that regulate transcriptional machinery by modifying histone proteins and chromatin structure. Histone acetylation, governed by histone acetyltransferases (HATs) and histone deacetylases (HDACs), plays a significant role in asthma pathophysiology. The dysregulation of the HAT/HDAC activity ratio contributes to airway inflammation and asthma severity (52), with HDAC2 being implicated in mediating the anti-inflammatory effects of corticosteroids. Reduced HDAC expression and activity have been observed in corticosteroid-resistant patients with severe asthma (53). Notably, restoring HDAC2 expression levels has been shown to restore corticosteroid sensitivity in asthmatic animal models (54).

The microbiome, a crucial factor influencing host health, is increasingly recognized as a significant contributor to asthma pathogenesis through its role in mediating epigenetic regulation. Epigenetic imprinting can determine the types of microorganisms that colonize the host, indicating a bidirectional relationship between epigenetics and gut microbiota that may jointly modulate asthma's pathogenesis and severity. This concept has led to the emergence of the “microbiota-metabolism-epigenetics axis” integrating metabolomic, metagenomic, transcriptomic, and epigenomic data to understand asthma's complexities (55). Healthy microbiota in the upper and lower airways are pivotal for maintaining organismal homeostasis. Recent studies have revealed distinctions between upper and lower airway microbiotas, with specific microbial enrichments and depletions observed in asthmatic bronchial brush samples (56). *Lactobacillus*, *Pseudomonas*, and *Rickettsia* were significantly enriched in bronchial brush samples from asthmatics, whereas *Prevotella*, *Streptococcus*, and *Veillonella* were significantly depleted (57). The relevance of upper vs. lower airway microbiota in asthma remains contentious, with increasing recognition that their interaction profoundly impacts asthma occurrence and development. Moreover, the gut-lung axis concept highlights the interplay between microbiota communities in the lung and gut, influencing immune responses and inflammation in asthma. Resveratrol, a plant-based polyphenol, has shown promise in alleviating airway inflammation and improving lung function in murine asthma models by modulating the gut-lung axis microbiota. Specifically, it can prompt enrichment of *Akkermansia muciniphila* and reduce the synthesis of LPS in lung and induce abundance of *Bacteroides acidifacien*s in gut (58).

Large-scale proteomics enables the elucidation of the functions and structures of approximately 30,000 proteins within a given cell, tissue, or organism. This approach is particularly useful for identifying disease-associated proteins and uncovering their dysfunctions and dynamic properties, which are integral to the heterogeneous nature of asthma and chronic obstructive pulmonary disease (COPD) (59). Mass spectrometry (MS) is extensively employed to quantitatively profile proteins and protein-protein interactions. Topological data analysis (TDA) of unbiased label-free quantitative MS has enhanced population stratification in asthmatic patients with granulocytic inflammation, identifying candidate protein biomarkers that may serve as novel therapeutic targets (60). Liu et al. established a murine model of steroid-resistant asthma exacerbation and conducted proteomics analysis to identify asthma-associated dysfunctional proteins, which were found to be enriched in MHC-I antigen presentation and IFN signaling pathways (61). They further validated signal transducer and activator of transcription-1 (STAT1) protein was the most significantly altered protein that contribute to steroid resistance in the pathogenesis of exacerbation, which can be considered as a promising therapeutic target.

Due to the heterogeneity of asthma, traditional transcriptome sequencing technologies, which are based on bulk blood or tissue samples, yield macroscopic results representing the average expression levels of genes across entire cell populations. This approach fails to capture the gene expression differences among individual cell types, thereby limiting our understanding of the pathogenesis and drug efficacy in heterogeneous respiratory diseases. Single-cell RNA sequencing (scRNA-seq) has emerged as a revolutionary technology that amplifies and sequences the whole transcriptome at the single-cell level, providing mRNA expression information specific to individual cell types. This allows for the identification of cell subpopulations and their associated identity markers in mice model and human, unveiling the heterogeneity of gene expression between cells and identifying rare cell types. scRNA-seq offers a novel and comprehensive approach to research, enabling the mapping of expression profiles of cells across the entire lung, constructing cell lineage tracing, and understanding the pathogenesis of asthma from multiple angles and levels in an intuitive manner (62). A pivotal study published in 2019 investigated and categorized cells in lung tissue, airways, and lymph nodes, highlighting the roles of various cell populations and their interactions in the development of asthma (63). Furthermore, Yang Ming et al. developed an LPS-induced steroid-resistant asthma exacerbation model in the context of an HDM-induced asthma model, followed by scRNA-seq to elucidate the characteristics of lung immune cells that drive steroid-resistant asthma exacerbation. This study revealed the correlation between an abnormally active immune response and the poor efficacy of glucocorticoid treatment, providing valuable insights for guiding precision medicine in asthma treatment (64).

The integration of multi-omics profiling provides a powerful opportunity for the systematic characterization of the molecular and biological profiles of asthma. Machine learning techniques have been theoretically applied to integrate multi-omics data from genomics, transcriptomics, epigenomics, proteomics, metabolomics, and the microbiome. This integration is instrumental in establishing models for investigating diagnostic and predictive factors across various sample populations and levels of asthma severity. Moreover, machine learning can be employed to define asthmatic endotypes and predict the efficacy of endotype-specific treatments, thereby facilitating decision-making in personalized precision medicine. Typically, the application of machine learning to omics data for patient stratification involves both unsupervised clustering techniques, such as K-means clustering, and supervised clustering methods, including random forests, decision trees, logistic regression, and feature selection. These approaches are crucial for identifying patterns and associations within complex datasets (65). However, multi-omics integrated analysis in asthma is still in its infancy, and the predicted biomarkers and risk factors of asthma require more effort to be verified by experimental examination and clinical trials. To achieve solid evidence, a large sample volume and improvement of the computational approach are necessary.

## Integration of multi-omics data facilitates novel patient stratification and personalized treatment approaches

Integrating multi-omics approaches with clinical characteristics, a heightened precision in asthma stratification and the realization of personalized medicine become attainable. For instance, a comprehensive analysis of protein abundance in plasma samples from adult asthma patients identified 115 proteins significantly associated with asthma (66). Among these, 20 proteins were found to be specifically linked to the asthma-atopy phenotype, which presents novel potential biomarkers for diagnostic purposes and therapeutic targeting in the management of asthma. Schofield et al. conducted a mass spectrometry (MS) analysis of the sputum proteome from 206 asthma patients, categorizing them into 10 clusters with distinct proteomic characteristics. This study offered novel insights into the mechanisms driving granulocytic and non-granulocytic inflammation in asthma, significantly aiding the prediction and management of treatment responses (60). In 2022, a comparative analysis of gene expression patterns between patients with mild-moderate and severe asthma identified five key regulators implicated in asthma development. The hub genes, including Wilms’ tumor 1 (WT1), zinc finger E-box binding homeobox 1 (ZEB1), arginine-glutamic acid dipeptide repeats (RERE), FOS like 1 (FOSL1), and miR-20a, were instrumental in-patient classification (67). Additionally, an epigenomic study of airway epithelial cells stratified asthma patients into four subpopulations based on differentially methylated CpG sites (68). These subpopulations were characterized by varying levels of eosinophilic infiltration in bronchoalveolar lavage fluid (BALF), which correlated with fractional exhaled nitric oxide levels and distinct clinical outcomes following inhaled corticosteroid treatment. More recently, the characteristics of sputum lipidomes have been found to differ significantly between asthma patients and healthy controls. Additionally, these lipid profiles, reflecting the types and levels of granulocytic inflammation cell-derived extracellular vesicles, exhibited considerable heterogeneity among asthma patients themselves and are useful for asthma stratification (69).

Microbiome studies on patients with severe asthma have revealed significant associations between specific bacterial taxa and inflammatory profiles. The relative abundance of *Proteobacteria phyla*, *Streptomyces*, and *Propionicimonas* was positively correlated with eosinophilia, whereas *Haemophilus* and *Moraxellagenera* were predominant in refractory neutrophilic asthma. These findings suggest that pathogenic bacteria can serve as novel biomarkers and therapeutic targets for the management of uncontrollable asthma (70, 71). In 2019, researchers employed 16S rRNA sequencing to characterize airway microbiota associated with different asthma endophenotypes. They found that *Trichoderma* was linked to airway inflammation in Th2-high asthma, while *Penicillium* was enriched in Th2-low asthma (72). They further employed a random forest tree model with selected key fungal classifiers that were associated with clinical parameters (e.g., forced expiratory volume, BALF cells, and oral corticosteroid use) to predict subtypes of asthma with 75% accuracy for BALF samples and 80% for endobronchial brush samples, highlighting different asthmatic endophenotype can be partitioned by distinct microbiota communities (72).

Qin et al. evaluated the therapeutic effect of the Jia-Wei Bu-Shen-Yi-Qi formula (JWBSYQF) treatment by proteomics analysis on a murine model of OVA-induced asthma and provided evidence to support JWBSYQF might improve lung function and reduce airway inflammation by blocking IL-17 signaling in severe asthma (73). Several genome-wide association studies (GWAS) had detected genetic variation in β2-adrenoceptor (ADRB2) was associated with long-acting β2 adrenoceptor agonists response and the increased risk of asthma exacerbation in pediatric asthma patients (74, 75). Hence, there are ongoing clinical trials investigating the therapeutic effects of ADRB2-guided and usual treatment on ADRB2-stratified pediatric asthma patients (76). Bromodomain and extra-terminal (BET) protein is an epigenetic reader regulating gene transcription by modifying chromatin remodeling and recognizing histone acetylated histones, which has been demonstrated to serve as modulators via directly mediating Th17 response in asthma (77). Nadeem et al. found BET Inhibitors could effectively reduce steroid resistances by restoring the expression level of HDAC2 in a mixed granulocytic (eosinophilic and neutrophilic) mouse model of asthma featured with Th2/Th17 endophenotype (78). Compared to non-asthmatics, general HDAC activity was higher in asthmatic primary bronchial epithelial cells, and inhibition of HDAC can ameliorate the epithelial defective barrier impaired by Th2 cells via inducing expression of tight junctions (79). Considering BET and HDAC synergistically regulating similar gene expression and biological effects in disease, HDAC/bromodomain-containing protein 4 (BRD4) dual inhibitors emerged as novel epigenetic targets and opened avenues for combination therapy for asthma (80).

“Unbiased Biomarkers for the Prediction of Respiratory Disease Outcomes” (U-BIOPRED) is collaborated by multiple countries and research centers aiming at collecting large-scale datasets from multiple sources. The integration of omics-derived biomarkers with clinical information facilitates the phenotyping of severe asthma into more precise subpopulations, paving the way for the development of effective novel therapies (81). The largest study on asthma is called “Severe Asthma Research Program (SARP)” which performs a computational approach to investigate pathological features of omics data from different cohorts. It proposes four phenotypes of asthma, they are early-onset mild allergic asthma, early-onset allergic moderate-to-severe remodeled asthma, late-onset nonallergic eosinophilic asthma, and late-onset non-eosinophilic nonallergic asthma (42).

**Figure 2:**
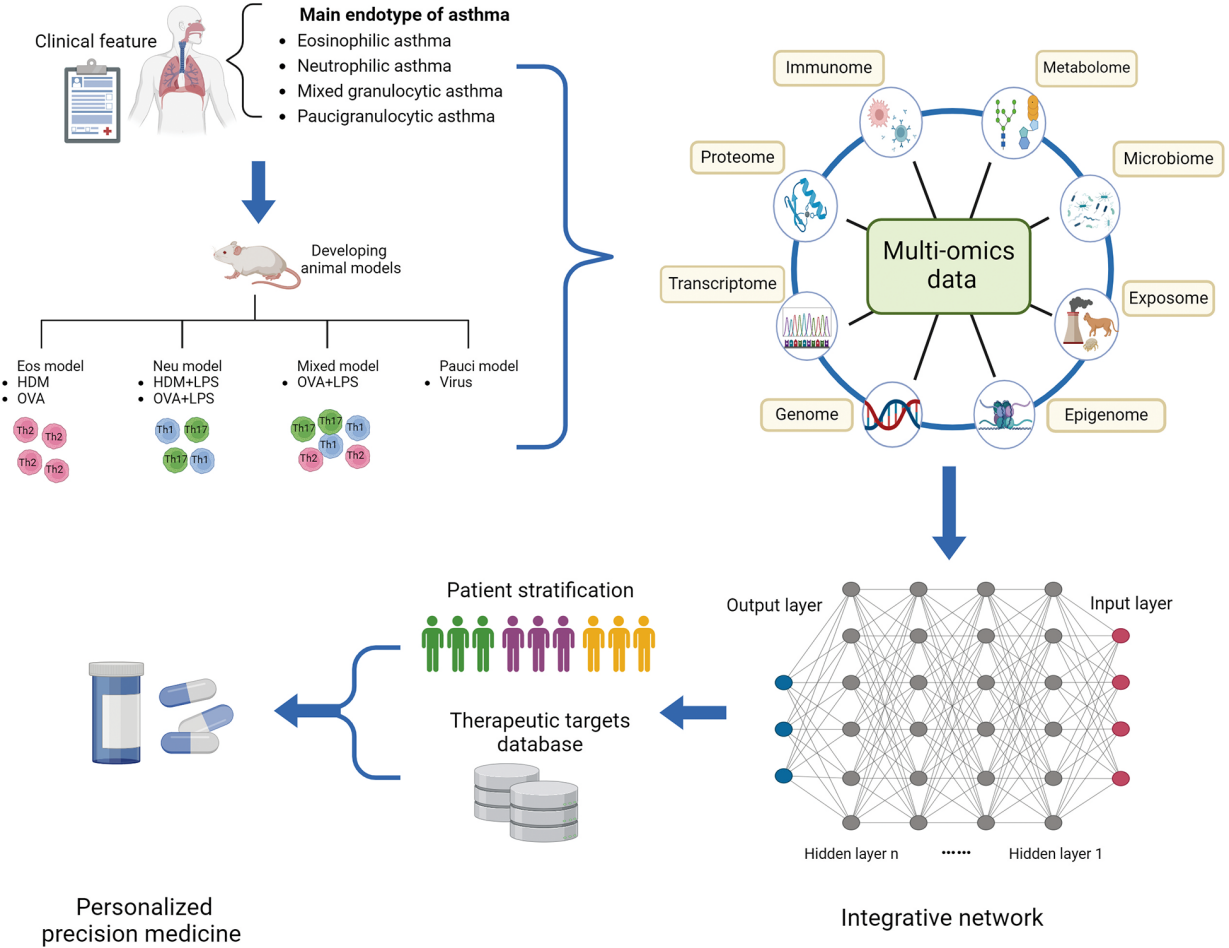
Multi-omics profiling to investigate the molecular mechanism underlying asthmatic pathogenesis and discover novel targets. Integrative multi-omics profiling on clinical specimens and murine models systematically deciphers distinct molecular and biological profiles of asthmatic endotype and underlying regulatory mechanisms driving a status. Machine learning improves the prediction sensitivity and accuracy of novel biomarkers and therapeutic targets for novel precision medicine. Eos, eosinophilic; HDM, house dust mite; LPS, lipopolysaccharide; Neu, neutrophilic; OVA, ovalbumin; Pauci, paucigranulocytic.

Omics technologies are contributing to a better understanding of the complex pathology of asthma and enable the development of accurate treatment options for distinct asthma endotypes (82). Recently, Global Initiative for Asthma (GINA) has provided additional recommendations in Step 5 for patients with difficult-to-treat or severe asthma to consider biologic therapies (83). Most asthma cases, particularly those with childhood or adolescent onset, are linked to IgE-mediated allergies; as a consequence, allergen immunotherapy targeting the IgE-mediated pathogenesis (omalizumab) has been proposed as a therapeutic selection (84). IL-4 and IL-13 were key regulators driving T2 inflammation; thus, targeting IL-4/IL-13 (dupilumab) or their receptors are particularly suitable for the asthmatics characterized by eosinophilic or allergic phenotype (85, 86). Besides, anti-IL-5 agents (reslizumab, mepolizumab, benralizumab) may be most effective for patients with eosinophil-predominant asthma, while anti-TSLP treatments are beneficial for both type 2 (T2) and non-T2 asthma (42). Defining asthma endotypes through comprehensive data, including clinical characteristics and biological biomarkers, will advance personalized management and precision-based care for asthma in the future (Figure 2).

## Conclusions

Asthma is one of the most serious chronic respiratory diseases, significantly impacting quality of life and imposing a substantial medical burden due to the lack of effective therapeutic options. The genetic and phenotypic heterogeneity of asthma complicates accurate patient stratification and treatment, particularly for those with severe asthma who often exhibit glucocorticoid resistance, posing a significant challenge for clinical management. High-throughput omics approaches—including genomics, epigenomics, transcriptomics, proteomics, metabolomics, and microbiomics—have emerged as novel technologies to systematically investigate the pathogenetic heterogeneity of diverse endophenotypes and elucidate the underlying molecular mechanisms. The integration of multi-omics approaches with machine learning is particularly promising, as it enables the identification of molecular and biological characteristics of distinct asthma subtypes and facilitates the screening of numerous therapeutic targets from multiple perspectives. This integrative approach enhances our capacity for precise patient classification and management. The development and refinement of integrative methods for analyzing large-scale multi-omics data are crucial for deriving meaningful diagnostic and predictive factors across diverse populations and varying asthma severities. This integration not only aids in defining asthmatic endophenotypes but also enhances the prediction of the effectiveness of endotype-specific therapies. In summary, the combination of basic and clinical research is essential for translating fundamental discoveries into clinical practice for the diagnosis, treatment, and management of asthma. This integrative approach holds the potential to substantially benefit patients by improving therapeutic strategies and outcomes.
